# ASSOCIATION OF AGE-FRIENDLY COMMUNITIES WITH INTRINSIC CAPACITY IN COMMUNITY-DWELLING OLDER ADULTS

**DOI:** 10.1093/geroni/igae098.0616

**Published:** 2024-12-31

**Authors:** Chunxiao Li, Kehan Liu, Wenting Peng, Yu Zheng, Chongmei Huang, Minhui Liu

**Affiliations:** Central South University, Changsha, Hunan, China (People’s Republic); Central South University, Changsha, Hunan, China (People’s Republic); University of Washington, Seattle, Washington, United States; The Third Xiangya Hospital of Central South University, Changsha, Hunan, China (People’s Republic); Ningxia Medical University, Yinchuan, Ningxia, China (People’s Republic); Ningxia Medical University, Yinchuan, Ningxia, China

## Abstract

Intrinsic capacity is crucial for older adults to maintain their functional independence and quality of life. Despite previous research have identified some environmental factors that impact intrinsic capacity, the associations between age-friendly communities and intrinsic capacity remained underexplored. To examine the relationship between the level of age-friendly communities and intrinsic capacity, in 2023, we conducted a cross-sectional study among 413 cognitively intact Chinese older adults from six age-friendly communities. Intrinsic capacity was measured with the Barthel Index (locomotion), Cornell Medical Index (vitality), Short-Form Mini-Nutritional Assessment (nutrition), Geriatric Depression Scale-15 (psychological health), and Mini-Mental State Examination (cognition). Community age-friendliness was assessed by the Age-Friendly Community Rating Scale. Covariates included demographics and health-related factors. Linear regression was used for analysis. These participants were aged between 65 and 94 years old, and male (52.06%). The level of age-friendly community was significantly associated with the intrinsic capacity of older adults (Standardized Beta = 0.776). The level of age-friendly community was associated with the intrinsic capacity scores in the unadjusted model (Beta = 0.861, P < 0.001) and the full-adjusted models (Beta = 0.609, P < 0.001). Additionally, the number of chronic diseases (Beta = -20.355, P < 0.001) and body mass index (Beta = -5.044, P < 0.05) were associated with lower scores of intrinsic capacity. Our findings suggest that the level of age-friendly communities is a protective factor for intrinsic capacity in older adults. Future research should focus on interventions to enhance community age-friendliness, thereby improving intrinsic capacity in community-dwelling older adults.

